# Prevalence, distribution, and social determinants of tobacco use in 30 sub-Saharan African countries

**DOI:** 10.1186/s12916-014-0243-x

**Published:** 2014-12-18

**Authors:** Chandrashekhar T Sreeramareddy, Pranil Mansingh Pradhan, Shwe Sin

**Affiliations:** Department of Population Medicine, Faculty of Medicine and Health Sciences, Universiti Tunku Abdul Rahman, Bandar Sungai Long, Selangor 43000 Malaysia; Department of Community Health Sciences, Patan Academy of Health Sciences (PAHS), P. O. Box 26500, Lagankhel-5, Lalitpur, Kathmandu, 44700 Nepal; Department of Preclinical Sciences, Faculty of Medicine and Health Sciences, Universiti Tunku Abdul Rahman, Bandar Sungai Long, Selangor 43000 Malaysia

## Abstract

**Background:**

Although the Framework Convention on Tobacco Control prioritizes monitoring of tobacco use by population-based surveys, information about the prevalence and patterns of tobacco use in sub-Saharan Africa is limited. We provide country-level prevalence estimates for smoking and smokeless tobacco (SLT) use and assess their social determinants.

**Methods:**

We analyzed population-based data of the most recent Demographic Health Surveys performed between 2006 and 2013 involving men and women in 30 sub-Saharan African countries. Weighted country-level prevalence rates were estimated for ‘current smoking’ (cigarettes, pipe, cigars, etc.) and ‘current SLT use’ (chewing, snuff, etc.). From the pooled datasets for men and women, social determinants of smoking and SLT use were assessed through multivariate analyses using a dummy country variable as a control and by including a within-country sample weight for each country.

**Results:**

Among men, smoking prevalence rates were high in Sierra Leone (37.7%), Lesotho (34.1%), and Madagascar (28.5%); low (<10%) in Ethiopia, Benin, Ghana, Nigeria, and Sao Tome & Principe; the prevalence of SLT use was <10% in all countries except for Madagascar (24.7%) and Mozambique (10.9%). Among women, smoking and SLT prevalence rates were <5% in most countries except for Burundi (9.9%), Sierra Leone (6%), and Namibia (5.9%) (smoking), and Madagascar (19.6%) and Lesotho (9.1%) (SLT use). The proportion of females who smoked was lower than SLT users in most countries. Older age was strongly associated with both smoking and SLT use among men and women. Smoking among both men and women was weakly associated, but SLT use was strongly associated, with education. Similarly, smoking among men and women was weakly associated, but SLT use was strongly associated, with the wealth index. Smoking and SLT use were also associated with marital status among both men and women, as well as with occupation (agriculturists and unskilled workers).

**Conclusions:**

Prevalence of smoking among women was much lower than in men, although the social patterns of tobacco use were similar to those in men. Tobacco control strategies should target the poor, not/least educated, and agricultural and unskilled workers, who are the most vulnerable social groups in sub-Saharan Africa.

## Background

Tobacco use has been long known to be a major cause of premature mortality [1] and has been attributed to cause 9% of all deaths worldwide [2]. Each year, an estimated 5.7 million deaths, 6.9% of years of life lost, and 5.5% of disability adjusted life years are caused by tobacco-related diseases [3]. The prevailing pattern of the tobacco epidemic could cause one billion deaths during the 21^st^ century and 80% of them could occur in low- and middle-income countries (LMICs) [4]. Though recent global estimates have shown a decreasing trend of smoking among both men and women, in 2012 there remained an estimated 967 million smokers living in 187 countries, with the number being expected to increase as the population grows [5].

The Framework Convention on Tobacco Control (FCTC) adopted in 2003 has been ratified by 177 countries worldwide [6]. Under the FCTC, monitoring of tobacco use worldwide by population-based surveys has been prioritized to understand disease patterns, assess the impact of tobacco control measures, and to assist tobacco control policy changes [7]. Major steps in this direction are the Global Tobacco Surveillance system [8], World Health Organization’s STEPS program [9], World Health Surveys (WHS) [10], and the International Tobacco Control (ITC) policy evaluation project [11] carried out in a number of countries spanning all continents. Nevertheless, data from these surveys do not comprehensively reflect tobacco use estimates, patterns, and types of tobacco products consumed in sub-Saharan Africa (SSA). For example, smokeless tobacco (SLT) products commonly consumed in South and South-East Asia [12] have health effects that are different from those of smoking [13,14] and are usually not emphasized much in tobacco control policies. This assumes great importance since the type of tobacco products consumed not only varies across countries [15] and regions [5], but also by age, gender, education, and economic status [16-19].

Among the multi-country surveys, tobacco use data from 14 SSA countries is available from the WHS [10] and Nigeria and Uganda in GATS [20], but none from ITC projects [11]. Further, the WHS and ITC projects focus on cigarette smoking only [11,17]. Information about tobacco use gathered from Demographic and Health Surveys (DHSs) performed on nationally representative samples of men and women can provide national-level estimates for each country and study the social distribution of tobacco use and type of tobacco products consumed in a particular region [15]. Pampel has provided estimates of cigarette smoking and its social determinants from DHSs performed in 14 SSA countries during the year 2006 or prior [21]. However, Pampel’s study does not provide estimates of SLT use and distribution of tobacco use by economic (wealth) status [21]. A systematic review of studies on adult tobacco use prior to the year 2005 in 14 SSA countries showed that the information varied due to the heterogeneity of the included studies [22]. The scale and pattern of the tobacco epidemic currently prevailing in the SSA countries is not clearly known except for some reports based on DHSs from Ghana [23] and Madagascar [24] and a national survey from Mozambique [25]. Inclusion of tobacco use questions in 30 countries’ DHSs during recent years provides a clear picture of the tobacco epidemic in the SSA region [26]. We aim to provide country-level prevalence estimates for smoking and SLT use and assess their social distribution (determinants) in 30 SSA countries.

## Methods

### Data source

We performed retrospective, secondary data analyses of the most recent DHSs, which are nationally representative, cross-sectional, household surveys. DHSs aim to provide reliable data on fertility, family planning, health and nutrition, health services utilization, health knowledge, and behaviors in more than 85 LMICs. DHSs are conducted by in-country/local institutions with funding from the United States Agency for International Development and technical assistance from the Opinion Research Corporation (ORC) Macro International Inc., Calverton, Maryland, USA [27]. The original microdata sets of the DHSs which had collected data about tobacco use in 30 SSA countries between 2006 and 2013 were downloaded from the DHS program [28] with their written permission. DHSs select households by two-stage stratified cluster sampling designs and usually oversampling is performed in the less populated provinces. In general, the DHS sampling method identifies clusters from both urban and rural areas by the probability proportional to size technique followed by a random selection of households from within the selected clusters. The head of each selected household answers all general questions about the household and lists the household members who reside there. Trained interviewers collect the data from all eligible men and women aged 15–49 years (in many countries men aged up to 64 years were interviewed) according to standard protocols using pretested questionnaires in local languages and their supervisors ensure that guidelines are adhered to for quality control and minimizing non-response [26,27]. The survey characteristics of DHSs from 30 countries included for our analyses are provided in Table 1.

**Table 1:** **Survey characteristics, sample sizes, and response rates for men and women participants of Demographic and Health Surveys in 30 countries in sub-Saharan Africa**

**Country**	**Dates field work**	**Households**	**Women**	**Men**	**Overall response rate (%)**
**EASTERN AFRICA**					
**1. Burundi**	August 2010 – January 2011	8,596	9,389	4,280	95.5
**2. Comoros**	August 2012 – December 2012	4,482	5,329	2,167	94.7
**3. Ethiopia**	December 2010 – May 2011	16,702	16,515	14,110	93.2
**4. Kenya**	November 2008 – February 2009	9,057	8,444	3,465	94.1
**5. Madagascar**	November 2008 – August 2009	17,857	17,375	8,586	94.4
**6. Malawi**	June 2010 – November 2010	24,825	23,020	7,175	95.1
**7. Mozambique**	June 2011 – November 2011	13,919	13,745	4,035	98.9
**8. Rwanda**	September 2010 – March 2011	12,540	13,671	6,329	98.9
**9. Tanzania**	December 2009 – May 2010	9,623	10,139	2,527	95.2
**10. Uganda**	June 2011 – December 2011	9,033	8,674	2,295	89.4
**11. Zambia**	April 2007 – October 2007	7,164	7,146	6,500	94.3
**12. Zimbabwe**	September 2010 – March 2011	9,756	9,171	7,480	89.5
**WESTERN AFRICA**					
**13. Benin**	December 2011 – March 2012	17,422	16,599	5,180	94.4
**14. Burkina Faso**	May 2010 – January 2011	14,424	17,087	7,307	97.7
**15. Cote d’Ivoire**	December 2011 – May 2012	9,686	10,060	5,135	91.0
**16. Ghana**	September 2008 – November 2008	11,778	4,916	4,568	95.4
**17. Liberia**	December 2006 – April 2007	6,824	7,092	6,009	92.5
**18. Mali**	November 2012 – February 2013	10,105	10,424	4,399	96.4
**19. Niger**	February 2012 – June 2012	10,750	11,160	3,928	93.5
**20. Nigeria**	February 2013 – June 2013	38,522	38,948	17,359	94.9
**21. Senegal**	October 2010 – April 2011	7,902	15,688	4,929	91.2
**22. Sierra Leone**	June 2013 – October 2013	12,629	16,658	7,262	91.8
**CENTRAL AFRICA**					
**23. Cameroon**	January 2011 – August 2011	14,214	15,426	7,191	96.4
**24. Congo (Brazzaville)**	September 2011 – February 2012	11,632	10,819	5,145	97.4
**25. Congo (Democratic)**	August 2013 – February 2014	18,171	18,827	8,656	98.0
**26. Gabon**	January 2012 – May 2012	9,755	8,422	5,654	97.5
**27. Sao Tome & Principe**	September 2008 – January 2009	3,536	2,615	2,296	84.5
**SOUTHERN AFRICA**					
**28. Lesotho**	October 2009 – January 2010	9,391	7,624	3,317	95.6
**29. Namibia**	May 2013 – September 2013	9,200	9,804	3,915	92.6
**30. Swaziland**	July 2006 – February 2007	4,843	4,987	4,156	89.6

### Outcome variable

We constructed a nominal outcome variable as ‘current smoking’ (cigarettes, pipe, cigars, etc.) and ‘current SLT use’ (chewing, snuff, etc.) based on responses provided to four main questions about tobacco use asked in both men’s and women’s questionnaires. The questions adopted were fairly similar in structure except for the response options in some countries. The respondents were asked four questions for which ‘yes’ or ‘no’ responses were available for the first two. A general outline of the questions is as follows:

1. Do you currently smoke cigarettes? Yes/No
2. Do you currently smoke or use any other type of tobacco? Yes/No
3. What (other) type of tobacco do you currently smoke or use? (pipe, chewing tobacco, snuff, etc.)
4. Over the past 24 hours, how many cigarettes have you smoked? (response as a numerical)

### Social variables

To study the social distribution of tobacco use, we used age (in single years), religion (classified as Islam, Catholic, Protestant, other Christian, and other/no religion), marital status (classified as ‘not in union’, ‘married’, ‘living together’, and ‘single’ which includes separated, widowed, and divorced), place of residence (classified as ‘rural’ and ‘urban’), current occupation (‘unemployed’, ‘professional’, ‘agriculture’, and ‘unskilled/manual work’), educational level (‘no education’, ‘primary’, ‘secondary’, and ‘higher’) and household wealth index. Wealth index is a reliable proxy indicator for economic status and it is calculated based on a standard set of household assets, dwelling characteristics, and ownership of consumer items as observed by the interviewer [29]. Each household is classified into quintiles where first quintile is the poorest 20% of the households and fifth quintile is the wealthiest 20% of the households [30].

### Ethics statement

Institutional review boards of ORC Macro International Inc. and in-country institutions which implemented the survey in each country provided ethical clearance for DHSs. The interviewers explained the survey details, voluntary participation, and confidentiality of information collected to each participant. Informed consent was obtained from each participant. No identifiable personal information was collected during the survey and data was archived by the DHS program.

### Data analysis

Prevalence rates of smoking and SLT use were estimated for each country, separately for men and women. For each country, overall weighted prevalence rates were calculated by including sample weights to account for complex sampling design adopted in DHSs. We pooled data from all 30 countries for men and women separately and calculated the weighted prevalence rates of smoking and SLT use by social factors; age groups, religion, place of residence, marital status, current occupation, education, and wealth quintiles. All weighted prevalence estimates were calculated using the ‘*svy*’ command in Stata intercool 10.0. To assess the social determinants of smoking and SLT use among men and women, logistic regression analyses were performed using country of domicile as dummy-variable controls and including within-country sample weight for each country into the regression models. For logistic regression analyses, we used the ‘*complex samples analysis*’ option in SPSS (Statistical Package for Social Sciences) to account for stratified, two-stage cluster sampling design used in DHSs.

## Results

### Sample characteristics

DHSs primarily aim to assess the indicators of maternal and child healthcare, resulting in larger women’s sample sizes in all included SSA countries (Table 1). Overall response rates were above 90% in most countries. In Western Africa, men were not asked about SLT use in Burkina Faso (Table 2). Information regarding religion was not collected in Tanzania and Niger. From the pooled data, more than half of the male and female respondents were aged under 30 years. More than half of the men and two-thirds of the women had not received any education or were educated up to primary level only. Both male and female respondents were almost evenly distributed across the wealth quintiles. About a quarter of both men and women were Muslims and about 60% of men were affiliated to Catholic, Protestant, or other Christian denominations. Overall, the highest proportion of respondents was married but 40.6% of men and 25.9% of women were ‘never in union’. Male respondents were mostly involved in agriculture (42.2%), whereas females were mostly unemployed (35.2%; Table 3).

**Table 2:** **Weighted prevalence estimates (95% confidence intervals) of smoking (cigarettes, pipe, and others) and smokeless tobacco use (chewing tobacco, snuff) among men and women of 30 countries in sub-Saharan Africa**

	**MEN**		**WOMEN**	
**Country (survey year)**	**Smoking**	**SLT use**	**Smoking**	**SLT use**
**EASTERN AFRICA**				
**Burundi (2011)**	21.24 (19.75, 22.73)	0.03 (−0.03,0.08)	9.89 (9.02, 10.75)	0.31 (0.18, 0.44)
**Comoros (2012)**	18.83 (16.67, 20.99)	7.72 (5.91, 9.55)	1.72 (1.22, 2.21)	2.99 (2.15, 3.85)
**Ethiopia (2011)**	6.75 (5.89, 7.61)	1.94 (1.47, 2.41)	0.57 (0.37, 0.77)	0.20 (0.09, 0.30)
**Kenya (2008)**	18.65 (16.42, 20.88)	2.05 (1.28, 2.83)	0.35 (0.21, 0.48)	1.29 (0.61, 1.97)
**Madagascar (2009)**	28.54 (27.15, 29.94)	24.66 (23.00, 26.32)	1.56 (1.18, 1.95)	19.63 (17.86, 21.40)
**Malawi (2010)**	17.73 (16.49, 18.98)	0.47 (0.27, 0.68)	0.38 (0.27, 0.49)	0.80 (0.62, 0.97)
**Mozambique (2011)**	20.73 (19.02, 22.43)	10.94 (9.62, 12.25)	2.76 (2.29, 3.23)	0.82 (0.59, 1.05)
**Rwanda (2011)**	12.91 (11.96, 13.86)	5.80 (5.14, 6.47)	1.01 (0.83, 1.19)	2.73 (2.39, 3.07)
**Tanzania (2010)**	19.60 (17.57, 21.62)	2.03 (1.27, 2.80)	0.54 (0.35, 0.73)	0.83 (0.56, 1.10)
**Uganda (2011)**	14.24 (12.42, 16.05)	2.94 (1.93, 3.96)	1.21 (0.89, 1.52)	1.50 (0.89, 2.11)
**Zambia (2007)**	24.10 (22.51, 25.68)	0.30 (0.16, 0.43)	0.81 (0.57, 1.05)	1.21 (0.83, 1.58)
**Zimbabwe (2011)**	21.99 (20.63, 23.35)	1.59 (1.20, 1.99)	0.16 (0.09, 0.24)	0.38 (0.23, 0.52)
**WESTERN AFRICA**				
**Benin (2012)**	8.47 (7.55, 9.38)	4.92 (4.17, 5.68)	0.28 (0.19, 0.36)	0.66 (0.52, 0.81)
Burkina Faso (2011)*	21.24 (19.97, 22.51)	–	0.09 (0.04, 0.14)	3.86 (3.22, 4.48)
**Cote d’Ivoire (2012)**	24.48 (22.67, 26.29)	0.61 (0.34, 0.89)	0.39 (0.19, 0.60)	1.27 (0.97, 1.58)
**Ghana (2008)**	7.55 (6.69, 8.40)	1.33 (0.95, 1.72)	0.17 (0.05, 0.29)	0.20 (0.08, 0.32)
**Liberia (2007)**	15.48 (13.86, 17.10)	2.34 (1.49, 3.19)	0.93 (0.63, 1.24)	2.37 (1.68, 3.06)
Mali (2012)*	15.86 (14.63, 17.09)	5.06 (3.96, 6.16)	0.19 (0.01, 0.28)	1.03 (0.69, 1.38)
**Niger (2012)**	14.02 (12.49, 15.55)	4.55 (3.60, 5.49)	0.02 (0.01, 0.06)	2.30 (1.59, 3.01)
**Nigeria (2013)**	9.20 (8.56, 9.84)	3.78 (3.33, 4.23)	0.22 (0.15, 0.29)	0.46 (0.31, 0.60)
**Senegal (2011)**	14.85 (13.43, 16.26)	6.63 (5.20, 8.06)	0.19 (0.09, 0.30)	0.23 (0.12, 0.34)
**Sierra Leone (2013)**	37.68 (35.16, 40.20)	1.54 (0.99, 2.09)	6.06 (5.37, 6.74)	4.74 (3.97, 5.51)
**CENTRAL AFRICA**				
**Cameroon (2011)**	14.77 (13.71, 15.82)	1.94 (1.52, 2.36)	0.57 (0.41, 0.74)	0.94 (0.59, 1.28)
**Congo (Brazzaville) (2012)**	19.91 (18.02, 21.81)	8.30 (6.97, 9.63)	0.54 (0.35, 0.72)	1.54 (1.22, 1.86)
**Congo (Republic) (2013)**	20.68 (18.81, 22.55)	8.67 (7.19, 10.16)	0.99 (0.78, 1.22)	3.22 (2.88, 3.56)
**Gabon (2012)**	22.38 (20.11, 24.66)	0.48 (0.21, 0.75)	3.11 (2.49, 3.73)	0.34 (0.18, 0.51)
**Sao Tome & Principe (2009)**	5.39 (4.23, 6.55)	2.59 (1.57, 3.62)	0.77 (0.38, 1.16)	0.73 (0.34, 1.19)
**SOUTHERN AFRICA**				
**Lesotho (2010)**	34.14 (32.13, 36.14)	1.40 (0.94, 1.86)	0.34 (0.19, 0.49)	9.12 (8.37, 9.87)
**Namibia (2007)**	21.84 (20.03, 23.65)	1.83 (1.25, 2.40)	5.87 (5.04, 6.69)	2.31 (1.96, 2.65)
**Swaziland (2007)**	14.40 (13.00, 15.79)	2.81 (2.25, 3.37)	1.13 (0.78, 1.48)	1.03 (0.70, 1.35)

*Data about SLT use was not collected from men.

**Table 3:** **Distribution of study sample and weighted prevalence estimates of smoking and smokeless tobacco use according to social factors from a pooled data set of 30 countries in sub-Saharan Africa**

	**Men (n = 169,500)**			**Women (n = 354,927)**		
	**Number (%)**	**Smoking (95% Cl)**	**SLT use (95% Cl)**	**Number (%)**	**Smoking (95% Cl)**	**SLT use (95% Cl)**
**Overall prevalence**		**17.17 (16.84, 17.51)**	**4.47 (4.25, 4.69)**		**1.27 (1.19, 1.35)**	**1.84 (1.74, 1.94)**
**Age group**						
15–19	35,780 (21.1)	4.08 (3.78, 4.38)	1.06 (0.91, 1.22)	75,541 (21.3)	0.43 (0.34, 0.51)	0.41 (0.34, 0.48)
20–29	51,389 (30.3)	17.05 (16.54, 17.57)	2.94 (2.71, 3.18)	130,014 (36.6)	0.90 (0.81, 0.99)	1.04 (0.95, 1.13)
30–39	39,705 (23.4)	23.09 (22.48, 23.69)	5.24 (4.88, 5.60)	91,373 (25.7)	1.58 (1.45, 1.71)	2.34 (2.18, 2.50)
40–49	27,541 (16.2)	23.81 (23.07, 24.56)	7.58 (7.11, 8.06)	57,999 (16.3)	2.75 (2.53, 2.96)	4.78 (4.49, 5.06)
>50^¶^	15,085 (8.9)	20.96 (20.10, 21.82)	10.31 (9.60, 11.02)	–	–	–
**Education**						
No education	35,592 (21.0)	19.88 (19.14, 20.62)	7.13 (6.67, 7.60)	117,279 (33.0)	1.88 (1.73, 2.03)	2.59 (2.40, 2.79)
Primary	59,935 (35.4)	19.27 (18.75, 19.79)	5.59 (5.22, 5.97)	124,961 (35.2)	1.03 (0.93, 1.12)	2.33 (2.17, 2.49)
Secondary	62,493 (36.9)	15.17 (14.71, 15.62)	2.69 (2.46, 2.92)	100,171 (28.2)	0.94 (0.81, 1.06)	0.71 (0.63, 0.79)
Higher	11,454 (6.8)	10.38 (9.66, 11.11)	1.37 (1.07, 1.67)	12,466 (3.5)	1.08 (0.81, 1.35)	0.13 (0.07, 0.20)
**Wealth index**						
Poorest	32,788 (19.3)	21.96 (21.22, 22.70)	7.76 (7.25, 8.27)	70,998 (20.0)	1.68 (1.51, 1.84)	3.52 (3.26, 3.78)
Poorer	31,136 (18.4)	19.09 (18.42, 19.76)	5.85 (5.41, 6.29)	66,794 (18.8)	1.40 (1.24, 1.56)	2.44 (2.25, 2.63)
Middle	31,877 (18.8)	17.67 (17.03, 18.31)	5.15 (4.71, 5.58)	66,309 (18.7)	1.21 (1.08, 1.34)	1.97 (1.80, 2.14)
Richer	33,743 (19.9)	15.79 (15.18, 16.40)	3.29 (2.97, 3.60)	69,322 (19.5)	1.08 (0.96, 1.20)	1.28 (1.13, 1.43)
Richest	39,956 (23.6)	13.41 (12.85, 13.97)	1.79 (1.54, 2.04)	81,504 (23.0)	1.09 (0.95, 1.23)	0.53 (0.44, 0.62)
**Religion** ^‡^						
Islam	40,417 (23.8)	16.41 (15.75, 17.07)	3.91 (3.54, 4.27)	88,552 (24.9)	0.97 (0.85, 1.08)	1.04 (0.90, 1.19)
Catholic	35,561 (21.0)	20.73 (20.07, 21.39)	5.83 (5.38, 6.29)	73,922 (20.8)	2.53 (2.28, 2.77)	2.60 (2.39, 2.81)
Protestant	27,573 (16.3)	16.62 (15.89, 17.36)	4.64 (4.13, 5.15)	59,540 (16.8)	1.57 (1.37, 1.77)	2.20 (1.98, 2.42)
Other Christian^†^	42,933 (25.3)	11.40 (10.90, 11.90)	2.24 (2.02, 2.46)	88,845 (25.0)	0.59 (0.52, 0.66)	1.21 (1.10, 1.33)
No religion*	16,374 (9.7)	29.46 (28.39, 30.52)	9.44 (8.68, 10.20)	22,189 (6.3)	1.86 (1.59, 2.14)	4.36 (3.89, 4.83)
**Marital status**						
Not in union	68,898 (40.6)	9.75 (9.40, 10.10)	1.58 (1.43, 1.73)	91,974 (25.9)	0.66 (0.57, 0.76)	0.45 (0.38, 0.51)
Married	76,839 (45.3)	20.96 (20.45, 21.47)	6.44 (6.09, 6.78)	189,266 (53.3)	1.12 (1.03, 1.21)	2.20 (2.06, 2.33)
Living together	16,722 (9.9)	23.16 (22.25, 24.08)	6.11 (5.51, 6.71)	42,064 (11.9)	2.35 (2.12, 2.59)	2.03 (1.82, 2.24)
Single^¥^	7,041 (4.2)	35.22 (33.69, 36.75)	7.87 (7.01, 8.74)	31,623 (8.9)	2.54 (2.28, 2.80)	3.62 (3.33, 3.92)
**Residence**						
Urban	59,640 (35.2)	15.79 (15.27, 16.31)	2.34 (2.11, 2.57)	121,955 (34.4)	1.23 (1.11, 1.36)	0.73 (0.64, 0.81)
Rural	109,860 (64.8)	17.99 (17.55, 18.42)	5.73 (5.41, 6.04)	232,972 (65.6)	1.29 (1.19, 1.39)	2.47 (2.32, 2.61)
**Occupation**						
Unemployed	26,407 (15.6)	6.83 (6.39, 7.27)	1.04 (0.89, 1.19)	125,072 (35.2)	0.84 (0.75, 0.92)	0.86 (0.76, 0.95)
Professional^×^	26,906 (15.9)	14.56 (13.92, 15.21)	2.37 (2.11, 2.63)	72,130 (20.3)	1.19 (1.05, 1.33)	0.88 (0.77, 0.99)
Agriculture	71,494 (42.2)	19.96 (19.39, 20.52)	7.39 (6.96, 7.82)	104,653 (29.5)	2.12 (1.92, 2.31)	3.70 (3.46, 3.95)
Unskilled	44,693 (26.4)	20.69 (20.11, 21.26)	3.52 (3.24, 3.80)	53,072 (15.0)	0.94 (0.81, 1.07)	2.10 (1.90, 2.30)

Totals do not add up to total sample of men and women for some categories of social factors including number of respondents who smoke tobacco or use SLT due to missing values.

^¶^In most countries except Tanzania, Swaziland, Namibia, and Liberia men older than 49 years (up to 54, 59, or 64 years) were surveyed.

^†^Includes various Christian faiths such as Adventist, Pentecostal, Eglise, Zionist, etc.

*Includes traditional religions such as Vodoun in Benin, Animism, etc.

^‡^Information about religion was not collected in Tanzania and Niger.

^¥^Single includes widowed, divorced, separated, and not living together any longer.

^×^Professional includes technical, manager, clerical, and business or sales; unskilled/manual includes household and domestic work other than agriculture.

### Prevalence of smoking and SLT use among men

**Figure 1:**
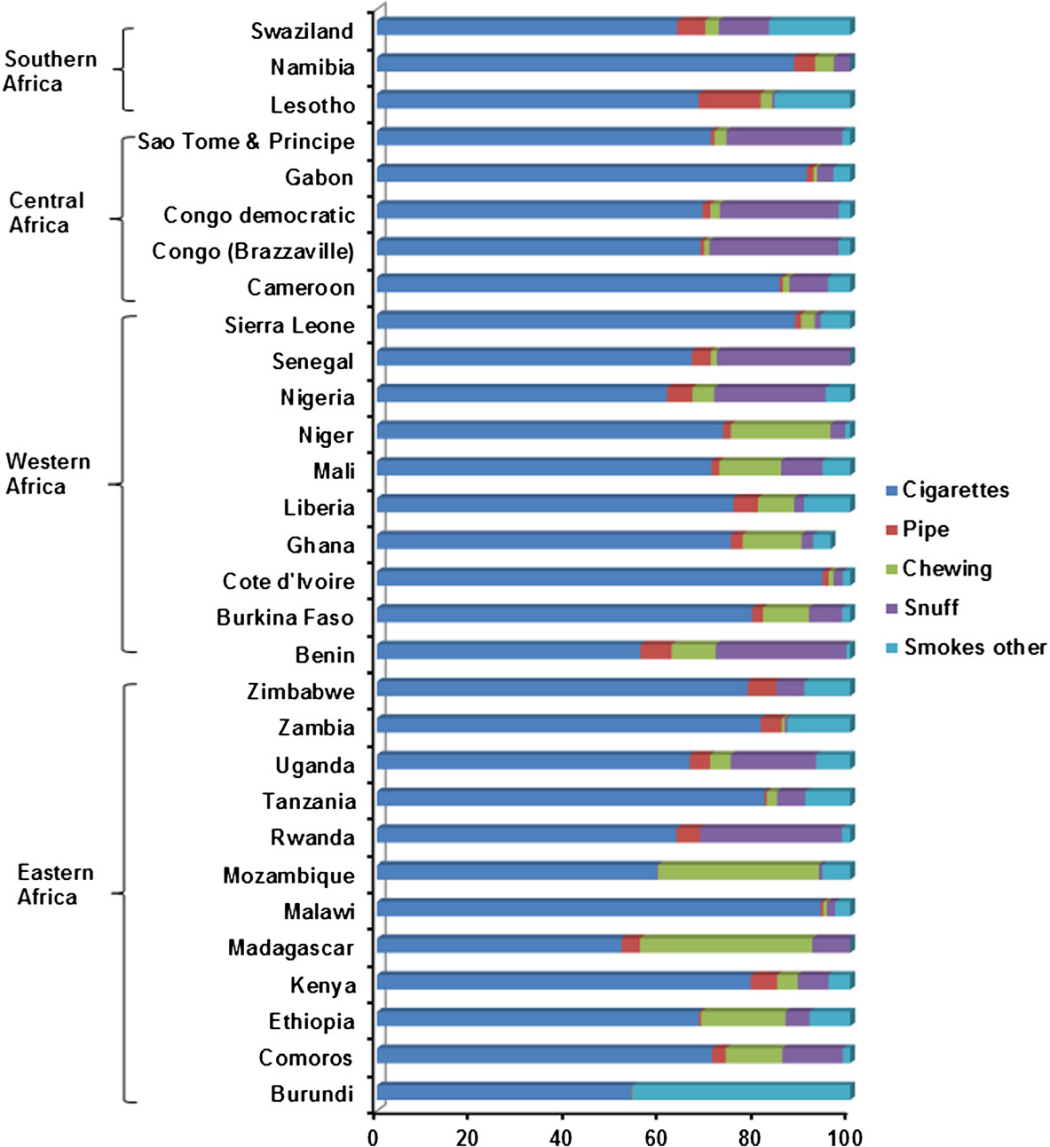
**Proportional distribution of various tobacco products consumed among tobacco-using men in 30 sub-Saharan African countries.** Percentage of respondents using multiple tobacco products was small and is not presented here.

In most East African countries, prevalence rates of smoking among men were similar, ranging from 12.91% (in Rwanda) to 24.10% (in Zambia), whereas Madagascar had the highest (28.54%) while Ethiopia had the lowest (6.75%) prevalence of smoking. In most East African countries, prevalence of SLT use among men was very low, ranging from 0.03% (in Burundi) to 7.72% (in Comoros) except for Madagascar, where prevalence was the highest (24.66%), followed by Mozambique (10.94%). In Western Africa, the prevalence rate of smoking among men was high in Sierra Leone (37.68%) and Cote d’Ivoire (24.48%) but low in Nigeria (9.20%) and Ghana (7.55%). In most West African countries the prevalence of SLT use among men was low, ranging from 0.61% in Cote d’Ivore to 6.63% in Senegal. In Central Africa, the prevalence of smoking among men was the highest in Gabon (22.38%) followed by the Democratic Republic of Congo (20.68%), whereas prevalence of SLT use was highest in the Democratic Republic of Congo (8.67%) followed by Congo (Brazzaville; 8.30%). In Southern Africa, Lesotho had the highest prevalence for smoking (34.14%), whereas the prevalence of SLT use among men was very low in most countries (1.40% to 2.81%; Table 2). Men who were using tobacco mostly smoked cigarettes in all SSA countries. However, men also consumed chewing tobacco (in Niger, Mozambique, Madagascar, Ghana, Ethiopia, and Burkina Faso) and snuff (in Uganda, Senegal, Sao Tome & Principe, Rwanda, Nigeria, Congo Brazzaville, and Benin; Figure 1).

### Prevalence of smoking and SLT use among women

**Figure 2:**
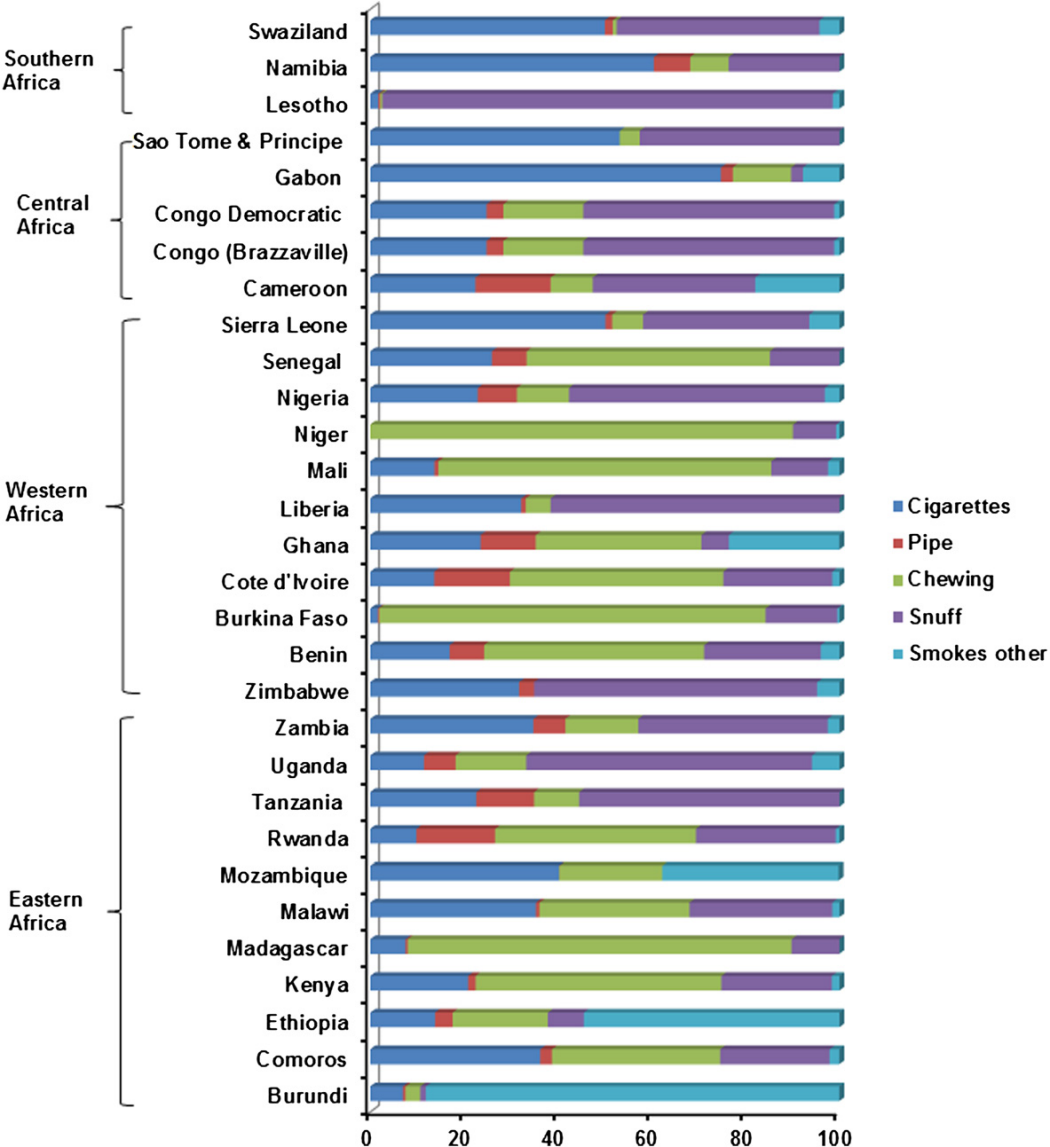
**Proportional distributions of various tobacco products consumed among tobacco-using women in 30 sub-Saharan African countries.** Percentage of respondents using multiple tobacco products was small and is not presented here.

In most East African countries, the prevalence of smoking and SLT use among women were very low (ranging from 0.16% to 2.76% for smoking and 0.20% to 2.99% for SLT use) except in Burundi (9.89%) for smoking and in Madagascar (19.63%) for SLT use. Similarly, in West Africa, the prevalence of smoking and SLT use were very low (ranging from 0.02% to 0.93% for smoking and 0.23% to 3.86% for SLT use) in most countries except in Sierra Leone (6.06% and 4.74%, respectively). In Central Africa, the highest prevalence of smoking among women was in Gabon (3.11%) and prevalence of SLT use was very low in most countries (0.34% to 3.22%). In Southern Africa, Lesotho had the highest prevalence of SLT use among women (9.12%) while Namibia had the highest prevalence for smoking among women (5.87%; Table 2). Women tobacco users in SSA countries mainly smoked cigarettes in Gabon, Swaziland, Sierra Leone, Namibia, and Sao Tome & Principe. However, in the remaining countries, a higher proportion of women tobacco users chewed tobacco in Niger, Madagascar, Burkina Faso, Congo, Cote d’Ivore, Benin, Rwanda, Kenya, and Senegal, while the proportion of those using snuff was higher in Lesotho, Zimbabwe, Uganda, Tanzania, Zambia, Nigeria, Liberia, Congo (Brazzaville), Swaziland, and Cameroon (Figure 2).

### Distribution of smoking and SLT use by social factors among men and women

Prevalence of smoking among rural men was higher (17.99% vs. 15.79%) and so was SLT use (5.73% vs. 2.34%; Table 3). Prevalence of both smoking and SLT use was higher among older men (aged ≥50 years) compared to the youngest (15–19 years). For example, prevalence of smoking was 4.08% among men aged 15–19 years while it was 23.81% among men aged 40–49 years and 20.96% among men aged 50 years or more. Similarly, among men, the prevalence of both smoking and SLT use was highest among poorest vs. the richest (21.96% vs. 13.41% for smoking; 7.76% vs. 1.79% for SLT use) and uneducated vs. highly education (19.88% vs. 10.38% for smoking; 7.13% vs. 1.37% for SLT use). There was a clear gradient across ordered variables wealth and education (Table 3). The prevalence of both smoking and SLT use was highest among single men (35.22% and 7.87%, respectively) while smoking prevalence was highest in agriculturists and unskilled/manual workers (19.96% and 20.69%, respectively) and SLT use was highest among agriculturists (7.39%). The prevalence of both smoking and SLT use was highest (29.46% and 9.44%, respectively) among men affiliated to other/traditional religions.

The prevalence of SLT use was much higher among rural compared to urban women (2.47% vs. 0.73%), although smoking prevalence was nearly equal (1.29% and 1.23%). The prevalence of both smoking and SLT use increased with age; the highest prevalence was among women aged 40–49 years (2.75% for smoking and 4.78% for SLT use). Similar to men, there was a gradient across the wealth and educational groups for smoking and SLT use among women (Table 3). The prevalence of both smoking and SLT use was highest among single women (2.54% and 3.62%, respectively) and among agriculturists (2.12% and 3.70%, respectively). Smoking prevalence was slightly higher (2.53%) among Catholic women, while SLT use was highest (4.36%) among women affiliated to other/traditional religions (Table 3).

### Association of smoking and SLT use with social factors among men and women

The association of smoking and SLT use with social determinants was assessed by multivariate analyses on separate pooled datasets for men and women from 30 countries. Smoking and SLT use were associated with age for both men and women. When compared to respondents aged 15–19 years, the odds of being a smoker and SLT user were 5- to 8-fold greater for those aged 40–49 years (>50 years for men; Table 4). Smoking among both men and women was weakly associated with education, whereas SLT use was strongly associated with education. Compared to men with a higher education, the odds of being a smoker and SLT user were 1.8- and 2.62-fold greater, respectively, for men who were uneducated. Similarly, compared to women with a higher education, the odds of being a smoker and SLT user were 2- and 11-fold greater, respectively, for women who were uneducated. Smoking among both men and women was weakly associated with wealth, whereas SLT use was strongly associated with wealth. Compared to the richest men, the odds of being a smoker and SLT user were 1.5- and 2.89-fold greater, respectively, for the poorest men. Compared to the richest women, the odds of being a smoker and SLT user were 1.24- and 3.36-fold greater, respectively, for the poorest women. Marital status was associated with smoking and SLT use among men. Compared to men and women who were not in union, the odds of being a smoker and SLT user were about 2-fold greater (adjusted odds ratios (aORs) varied from 1.48 to 2.07) for men and women who were single (separated, divorced, and widowed). Among both men and women, area of residence (urban/rural) was weakly associated with smoking but unassociated with SLT use (Table 4). Men’s occupation was associated (weakly) with smoking and SLT use but women’s occupation was associated with SLT use only. Compared to unemployed men, the odds of being a smoker and SLT user were nearly 2-fold greater for men doing unskilled or manual work. Religious affiliation was associated with smoking and SLT use among both men and women. The odds of being a smoker and SLT user were about 2-fold greater (aORs 1.56 to 2.48) for men who followed other/traditional religions whereas the odds of being a smoker were 2-fold greater (aOR 2.37) for women who followed other/traditional religions (Table 4).

**Table 4:** **Social determinants (from pooled data) of smoking and smokeless tobacco use among men and women of 30 countries in sub-Saharan Africa**

	**Men**				**Women**			
	**Smoking**	***P*** **value**	**SLT use**	***P*** **value**	**Smoking**	***P*** **value**	**SLT use**	***P*** **value**
	aOR (95% CI)		aOR (95% CI)		aOR (95% CI)		aOR (95% CI)	
**Age group**		<0.001		<0.001		<0.001		<0.001
15–19	1		1		1		1	
20–29	0.87 (0.81, 0.93)		1.22 (1.10, 1.34)		1.62 (1.47, 1.80)		1.98 (1.82, 2.15)	
30–39	0.91 (0.85, 0.97)		1.77 (1.60, 1.96)		2.58 (2.28, 2.91)		4.08 (3.70, 4.49)	
40–49	1.22 (1.14, 1.32)		2.93 (2.61, 3.29)		4.30 (3.36, 5.51)		8.37 (6.70, 10.44)	
≥50^¶^	5.31 (4.75, 5.93)		7.03 (5.76, 8.59)		–		–	
**Education**		<0.001		<0.001		<0.001		<0.001
Higher	1		1		1		1	
Secondary	0.90 (0.85, 0.95)		1.15 (1.05, 1.26)		1.80 (1.59, 2.04)		1.49 (1.36, 1.64)	
Primary	1.13 (1.06, 1.21)		1.75 (1.56, 1.96)		1.88 (1.60, 2.22)		2.97 (2.56, 3.46)	
No education	1.80 (1.61, 2.00)		2.62 (2.04, 3.36)		2.00 (1.47, 2.72)		10.68 (6.60, 17.26)	
**Wealth index**		<0.001		<0.001		<0.001		<0.001
Richest	1		1		1		1	
Richer	1.16 (1.10, 1.23)		1.30 (1.18, 1.43)		1.13 (1.00, 1.28)		1.33 (1.21, 1.47)	
Middle	1.23 (1.16, 1.31)		1.32 (1.18, 1.47)		1.27 (1.10, 1.46)		1.49 (1.34, 1.66)	
Poorer	1.38 (1.29, 1.48)		1.92 (1.68, 2.18)		1.37 (1.18, 1.58)		2.05 (1.79, 2.34)	
Poorest	1.51 (1.39, 1.64)		2.89 (2.40, 3.50)		1.24 (1.02, 1.50)		3.36 (2.71, 4.16)	
**Religion**		<0.001		<0.001		<0.001		<0.001
Islam	1		1		1		1	
Catholic	1.67 (1.53, 1.83)		1.62 (1.33, 1.99)		1.95 (1.49, 2.54)		1.52 (1.18, 1.96)	
Protestant	1.40 (1.30, 1.50)		0.88 (0.77, 0.99)		1.04 (0.87, 1.25)		0.77 (0.67, 0.90)	
Other Christian^†^	2.08 (1.92, 2.26)		1.11 (0.96, 1.28)		2.10 (1.70, 2.59)		0.99 (0.84, 1.16)	
No Religion*	2.48 (2.30, 2.67)		1.56 (1.34, 1.82)		2.37 (1.91, 2.94)		1.13 (0.95, 1.35)	
**Marital status**		<0.001		<0.001		<0.001		<0.001
Not in Union	1		1		1		1	
Married	1.66 (1.52, 1.82)		1.21 (1.02, 1.44)		1.11 (0.96, 1.28)		1.01 (0.88, 1.16)	
Living Together	1.93 (1.78, 2.09)		1.29 (1.12, 1.49)		1.58 (1.39, 1.80)		1.25 (1.13, 1.38)	
Single^¥^	2.07 (1.89, 2.26)		1.48 (1.26, 1.75)		2.04 (1.68, 2.49)		1.79 (1.48, 2.16)	
**Residence**		<0.001		0.878		<0.001		0.288
Urban	1		1		1		1	
Rural	1.12 (1.06, 1.19)		1.01 (0.90, 1.14)		1.52 (1.31, 1.76)		0.92 (0.79, 1.07)	
**Occupation**		<0.001		<0.001		0.051		<0.001
Unemployed	1		1		1		1	
Professional^×^	1.13 (1.07, 1.19)		0.85 (0.76, 0.93)		0.99 (0.82, 1.20)		0.88 (0.76, 1.02)	
Agriculture	1.37 (1.28, 1.46)		1.29 (1.13, 1.49)		1.02 (0.85, 1.23)		1.30 (1.10, 1.54)	
Unskilled	1.82 (1.68, 1.97)		1.71 (1.44, 2.04)		1.18 (0.99, 1.40)		1.11 (0.94, 1.30)	

Multivariate analyses were statistically controlled for age, education, wealth index, religion, marital status, type of residence, and occupation; aOR, Adjusted odds ratios; CI, Confidence interval; SLT, Smokeless tobacco.

^¶^In most countries men older than 49 years (i.e., up to 54, 59, or 64 years) were surveyed.

^†^Includes various Christian faiths such as Adventist, Pentecostal, Eglise, Zionist, etc.

*Includes traditional religions such Vodoun in Benin, Animism etc.

^¥^Single includes widowed, divorced, separated, and not living together any longer.

^×^Professional includes technical, manager, clerical and business or sales; unskilled/manual includes household and domestic work other than agriculture.

## Discussion

Our analyses of DHS data provided national-level estimates for tobacco use in 30 out of 47 SSA countries by sex and type of tobacco consumed. The data obtained highlights the scale of the tobacco epidemic and describes the pattern of smoking and SLT use according to social groups. Among men, the prevalence of smoking (mainly cigarettes) was very high relative to SLT use in all countries except Madagascar and Mozambique. Among all the SSA countries, smoking prevalence among men was high in Sierra Leone, Lesotho, and Madagascar, where nearly a third of adult men were current smokers. In most countries, the prevalence of both smoking and SLT use among women was very low compared to men and the highest prevalence of smoking and SLT use was found in Burundi and Madagascar, respectively. Compared to men, women were using more diverse tobacco products such as cigarettes, pipe, snuff, chewing tobacco, and other types.

Since most SAA countries are poor and have lower literacy rates, it is commonly thought that the prevalence of tobacco use is lower, resulting in a low priority for tobacco control. However, following the economic growth currently experienced in many SSA countries, it is estimated that smoking prevalence will increase [31]. The lower prevalence estimates for most SSA countries presented herein were comparable to those in Pampel’s study from 14 SSA countries [21]. A systematic review has also concluded that adult tobacco use prevalence in many SSA countries is lower than in developed and other developing countries [22]. The very small differences in prevalence estimates between our study and those of Pampel’s indicate that the prevalence has changed minimally or else the differences may have been solely due to sampling errors. Nevertheless, another DHS-based study reported that prevalence of smoking among Ghanaian men had decreased by 1.7% between 2003 and 2008 [23]. Further, the current smoking and SLT use prevalence in most of the SSA countries, except Madagascar, Sierra Leone, and Lesotho, was much lower than in South and South-East Asian countries [15]. However, the current smoking prevalence estimates presented herein cannot be compared with those in Nigeria and Uganda assessed by GATS [20] or with the WHS [17], which included 14 SSA countries, since these surveys defined current smoking as smoking any form of tobacco either daily or occasionally [17,19]. Moreover, our estimates are also different from those by Ng et al. [5], since the authors adopted a different definition of daily smoking and used comprehensive data sources and robust statistical analyses.

Overall, prevalence rates of smoking and SLT use among both men and women in 30 SSA countries were much lower than in South and South-East Asian countries [15] and other regions of the world [5,32]. A higher prevalence of SLT use among men was found in some SSA countries only, for example, chewing tobacco in Madagascar [24] and Mozambique [25], and snuff inhalation in Rwanda and Senegal. Prevalence of SLT use among women was very low in most SSA countries except in Madagascar (mainly chewing tobacco) [24] and Lesotho (mainly inhaling snuff), unlike the pattern in South Asian countries where both men and women used more diverse types of SLT products [15]. A higher prevalence of SLT use among men and women was reported in India, Pakistan, Nepal, and Bangladesh, but not in other South-East Asian countries [15]. Thus, the high prevalence of SLT use among men and women in Madagascar may be explained by a high proportion of Madagascans having South Asian descent where SLT use is very high [23]. Sierra Leone had the highest prevalence of smoking among men, consistent with a previous study [23]. However, of all SSA countries, Burundi had the highest prevalence (9.8%) of smoking among women. The lower prevalence of smoking and SLT use among women in most SSA countries is in accordance with an earlier study [19]. A positive gradient by age among both men and women for smoking has been previously reported [15,19,21,22]. This pattern may be explained by cohort effects, i.e., smoking was less likely to be initiated in more recent decades, or by age effects, i.e., respondents continued to initiate as they grew older. However, we could not assess whether the positive gradient by age was caused by cohort or age effects since we analyzed single cross-sectional survey data from each country. Previous studies have reported the existence of wealth-related inequalities in smoking [17] and the social determinants of tobacco use [19] in LMICs. These studies indicated that, in most LMICs, the poorest men and women were more likely to smoke than the richest, which is similar to our results. In SSA countries, SLT use was strongly associated with wealth, i.e., poorer men and women were more likely to use SLT, similar to the findings from South and South-East Asian countries [15]. It is thought that poorer people may consume tobacco to suppress their hunger [33] since many smokers believe that smoking has an appetite-suppressing effect; many tobacco companies have exploited this by introducing appetite suppressant additives to the cigarettes [34]. Compared to men and women with a higher education, uneducated men and women were more likely to smoke and use SLT, consistent with the results of previous studies from Africa [21] and other regions [19]. Less educated (illiterate) people may be more vulnerable to tobacco use as they lack knowledge about their adverse health effects [35] or else this pattern may be due to parental influence, peer pressure [36], and cultural acceptance [37].

Cigarette smoking has been reported to be higher among urban residents [21,38]; however, in our study, rural men and women in SSA countries were more likely to smoke. Our findings suggest that perhaps smokers who are usually poor and uneducated may be living in rural areas. In our study, Catholic, traditional religions, or no religious affiliation were associated with smoking and SLT use, which may likely be a residual confounder. Although none of the religions promote smoking or other unhealthy behaviors, there is no conclusive evidence for this negative relationship between religion and tobacco use [39]. As compared to those who were never in union, single men (separated, divorced, or widowed) were more likely to use tobacco; this was also the case for married women. These findings are not consistent with our previous studies or with others from Africa [15,21,23,40]. Our analyses show that agriculturists and unskilled or manual workers had a higher risk of smoking or SLT use, which is in accordance to a previous study in 14 SSA countries [21] and another in the United States of America [41]. Nevertheless, we agree that occupation, which reflects an individual’s social standing, is also related to an individual’s education and income [42] and therefore social standing affects health and health behaviors [43].

In general, the prevalence of smoking and SLT use was very low among women, in agreement with previous reports from SSA countries [21,22]. However, the higher prevalence of smoking among women in Burundi, Sierra Leone, and Namibia, and SLT use in Madagascar and Lesotho warrants gender-specific tobacco control interventions in these countries. Our analysis identified that poor, uneducated or less educated (up to primary school), agriculturists, and manual or unskilled workers as the most vulnerable groups. Research has shown that in lower socio-economic groups, who already have a scarce income, smoking may divert their resources from education, health care, housing, and quality food to purchase cigarettes [44]. Even though tobacco use is generally lower in SSA countries, they have a higher burden of nutrition and communicable disease [45] and may face an additional burden from non-communicable diseases [46] if timely action is not taken to curb this early-stage tobacco epidemic. To reduce the burden of tobacco-related ill health, interventions should be directed at eliminating its root causes such as illiteracy and poverty itself [17]. There is a need for the relevant authorities to act by addressing the disparities in tobacco use, failing which inequalities in health may widen further [16]. As of July 2014, 42 of the 47 SSA countries had ratified the FCTC, whose provisions include a ban on tobacco advertising, promotions, and partnerships, warning labels on tobacco product packages, measures to prevent exposure to second hand smoke, and increased taxation [7]. It is necessary that all SSA countries ratify the FCTC, especially Malawi and Mozambique, which have a higher smoking prevalence.

Analyses of DHSs provided a regional overview of the tobacco epidemic and such data may be utilized for monitoring the tobacco epidemic at country-level and assess prevalence by population subgroups. If DHSs in more countries collect tobacco use data, an updated analysis can provide a complete scenario of tobacco use in the SSA region. In a vast continent such as Africa, prevalence of tobacco use is lower than in other regions, but varies much across the 30 countries included herein. For example, smoking among men was 5.4% in Sao Tome & Principe but 37.7% in Sierra Leone. What can explain the between-country differences in prevalence of smoking and SLT use? Such inter-country variations may be explained by country characteristics such as the economy, i.e., gross domestic product, cultural factors, access of tobacco companies to sales, tobacco control policies, and pricing of tobacco products. Nevertheless, we did not study these factors as it was beyond the scope of this paper. Further research using country-level aggregate data about these factors and multi-level modeling may provide a better understanding about the reasons for inter-country variations in tobacco use.

Prevalence estimates retrieved from DHS data have some limitations due to the survey design and questions asked to assess tobacco use. DHSs have limited the age of men and women respondents from 15–64 and 15–49 years, respectively. Therefore, true population prevalence rates may be underestimated if the prevalence rates among older men (>64 years) and women (>49 years) were higher. The association between social factors and tobacco use lacks a temporal relationship due to the cross-sectional design of the DHSs. We could only estimate current smoking and current SLT use since limited information was collected about tobacco use. Tobacco use based on self-reports may have been underreported due to stigma, especially among the young and women, leading to misclassification bias and underestimation of prevalence rates. However, there was no means to verify self-reported tobacco use by estimating biomarkers such as urinary cotinine levels.

## Conclusions

The prevalence of smoking among women was much lower than among men, but showed similar social patterns. Tobacco control strategies should target the poor, not (least) educated, and agricultural and unskilled workers, who are the most vulnerable social groups in the SSA region. DHSs can provide reliable estimates for surveillance of tobacco use at country-level and by social groups. As most SSA countries are at the early stages of the tobacco epidemic, tobacco control efforts in Africa should focus on health promotion to stop the initiation of tobacco use in addition to cessation.

## Abbreviations

aORs: Adjusted odds ratios
DHSs: Demographic and Health Surveys
FCTC: Framework Convention on Tobacco Control
ITC: International Tobacco Control
LMICs: Low- and middle-income countries
ORC: Opinion Research Corporation
SSA: Sub-Saharan African
SLT: Smokeless tobacco
WHS: World Health Surveys
